# Nanoshield‐Assisted Viral Gene Therapy with Induction of Non‐Apoptotic Cell Death and Durable Antitumor Immunity

**DOI:** 10.1002/advs.202507550

**Published:** 2025-08-04

**Authors:** Soo‐Hwan Lee, Yunkyeong Cho, Seunghwan Bang, Daeun Sung, Jahyun Koo, Seoyoung Kim, Youngil Koh, Hojun Kim, Hyojin Lee

**Affiliations:** ^1^ Biomaterials Research Center Korea Institute of Science and Technology (KIST) Seoul 02792 Republic of Korea; ^2^ Division of Biomedical Science and Technology KIST School Korea University of Science and Technology Seoul 02792 Republic of Korea; ^3^ Center for Advanced Biomolecular Recognition Korea Institute of Science and Technology (KIST) Seoul 02792 Republic of Korea; ^4^ School of Biomedical Engineering Korea University Seoul 02841 Republic of Korea; ^5^ Interdisciplinary Program in Precision Public Health Korea University Seoul 02841 Republic of Korea; ^6^ Department of Internal Medicine Seoul National University Hospital Seoul 03080 Republic of Korea; ^7^ SKKU‐KIST Department of Integrative Biotechnology College of Biotechnology and Bioengineering Sungkyunkwan University Suwon Gyeonggi 16419 Republic of Korea

**Keywords:** adeno‐associated virus (AAV), cancer immune therapy, manganese‐nanoshield, necroptosis, receptor‐interacting kinase 3 (RIPK3)

## Abstract

Non‐apoptotic cell death have emerged as promising strategies to overcome apoptotic resistance in cancer therapy. We suggest a hybrid gene delivery platform integrating adeno‐associated virus (AAV)‐mediated expression of receptor‐interacting kinase 3(RIPK3) with manganese dioxide‐polyethyleneimine (MnO_2_‐PEI) to induce necroptosis and immunogenic cell death (ICD), thereby remodeling the tumor microenvironment and enhancing antitumor immunity. This platform combines high transduction efficiency with the tumor‐accumulation ability and immunostimulatory potential of non‐viral carriers. The MnO_2_–PEI nanosheets shields AAV from immune and hepatic clearance, thus enhancing tumor accumulation. This addresses a key limitation of naked AAV delivery. Simultaneously, the AAV payload offsets non‐viral systems’ low gene delivery efficiency. The platform induces robust damage‐associated molecular patterns (DAMP) and tumor antigen release, thereby promoting dendritic cell maturation and cytotoxic T cell infiltration. Furthermore, Mn²⁺‐induced reactive oxygen species (ROS) amplify ferroptosis and, in conjunction with RIPK3‐mediated necroptosis, remodel the immunosuppressive tumor microenvironment by promoting M1 macrophage polarization and a Th1‐type immune response. In tumor re‐challenge models, AAV/MnO_2_–PEI‐treated mice exhibited durable antitumor immunity, thereby highlighting the potential of platform to establish long‐term immune memory. This hybrid delivery system provides a potent strategy for synergistic cancer immunotherapy, effectively overcoming the limitations of both viral and non‐viral vectors.

## Graphical abstract

A novel hybrid gene delivery platform combines adeno‐associated virus (AAV)‐mediated receptor‐interacting kinase 3 (RIPK3) expression with manganese dioxide‐polyethyleneimine (MnO_2−_PEI) nanoshield to induce necroptosis, ferroptosis, and immunogenic cell death, effectively reprogramming the tumor microenvironment. This strategy synergizes high gene transduction efficiency with potent immune activation promoting dendritic cell maturation, T‐cell infiltration, and durable antitumor immunity, offering a promising approach for next‐generation cancer immunotherapy.

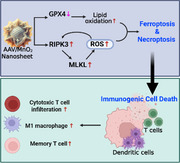

## 1 Introduction

In recent decades, the clinical paradigm of cancer treatment has transitioned from targeting mechanisms of cell survival and proliferation to inducing programmed cancer cell death.^[^ ^1^ ^]^ Apoptosis‐based strategies have dominated this landscape; however, the emergence of apoptotic resistance, therapeutic escape mechanisms, and reduced efficacy has highlighted the urgent need for alternative approaches.^[^ ^2^, ^3^ ^]^ In this context, non‐apoptotic cell death pathways such as necroptosis have garnered significant attention due to their ability to overcome resistance mechanisms in cancer cells.^[^ ^4^ ^]^ Unlike apoptosis, necroptosis is orchestrated by receptor‐interacting protein kinase 3(RIPK3), which activates mixed lineage kinase domain‐like pseudokinase (MLKL), leading to membrane rupture and release of damage‐associated molecular patterns (DAMPs).^[^ ^5^, ^6^, ^7^ ^]^ These DAMPs not only contribute to direct tumor cell elimination but also initiate potent antitumor immune responses by promoting dendritic cell (DC) maturation and creating a pro‐inflammatory tumor microenvironment (TME).^[^ ^8^ ^]^ However, the low endogenous expression of necroptotic mediators in many tumors necessitates exogenous gene delivery to achieve therapeutic activation.

Gene delivery platforms, including plasmid DNA, mRNA, and viral vectors, have emerged as pivotal tools in the modulation of cancer cell death pathways.^[^ ^9^ ^]^ Among these, adeno‐associated viruses (AAVs) are widely employed due to their high gene transduction efficiency and tissue specificity.^[^ ^10^, ^11^ ^]^ However, systemic AAV delivery is hindered by several key challenges, including immunogenicity, rapid hepatic clearance, and reduced transduction efficiency in tumors.^[^ ^12^, ^13^ ^]^ To overcome these limitations, AAV surface modification strategies, including polymeric and lipid‐based encapsulation, have been explored to improve delivery efficiency and reduce immune recognition.^[^ ^14^, ^15^ ^]^ However, these surface coatings primarily serve passive roles either shielding vectors from immune attack or enhancing intracellular trafficking. Consequently, a new generation of AAV surface engineering is required, in which the coating itself synergizes with gene payloads to potentiate anticancer effects.

Furthermore, reliance on gene expression as the sole inducer of non‐apoptotic cell death may prove inadequate, given the complexity and plasticity of tumor cell death pathways. A compelling strategy to amplify therapeutic responses is represented by co‐delivery of molecular cofactors. Reactive oxygen species (ROS) have been reported to act as amplifiers of the necroptotic response and may further contribute to cancer cell elimination.^[^ ^16^, ^17^ ^]^ Manganese dioxide (MnO_2_) nanosheets, which are recognized for their biocompatibility and large surface area, have shown great promise in biomedical applications including drug delivery.^[^ ^18^ ^]^ Upon interaction with intracellular glutathione (GSH), MnO_2_ nanosheets undergo decomposition into Mn^2+^ ions, thereby triggering a fenton‐like reaction that generates ROS.^[^ ^19^, ^20^, ^21^ ^]^ This process depletes Glutathione peroxidase 4 (GPX4), induces lipid peroxidation, and activates ferroptosis, a type of non‐apoptotic cell death that is closely related to necroptosis. The concurrent activation of ferroptosis and RIPK3‐mediated necroptosis through Mn^2+^‐induced ROS and RIPK3 expression creates a potent synergistic effect that not only enhances tumor cell killing but also reorganizes the tumor immune microenvironment. These dual mechanisms provide a robust foundation for the development of next‐generation cancer immunotherapies that harness the immunostimulatory potential of non‐apoptotic cell death.

In this study, MnO_2_ nanosheets, engineered as a nanoshield, were functionalized with branched PEI to form electrostatically complexes with AAV, thereby enhancing delivery efficiency while mitigating immunogenicity. The MnO_2_ nanosheet undergoes decomposition under intracellular reductive conditions, generation ROS that synergistically amplify RIPK3‐mediated necroptosis. The combination of this strategy with RIPK3‐expressing AAV constructs, shows to significantly enhance cancer immunotherapy through the coordinated induction of immunogenic cell death. This multimodal platform integrates virology and nanotechnology, thus advancing gene therapy by coupling cell death pathways with immune activation. By orchestrating these three processes – necroptosis, ferroptosis, and immune stimulation – in a simultaneous manner, this approach offers a transformative therapeutic paradigm with strong translational potential in the field of oncology.

## 2 Results and Discussion

### 2.1 Characterization of AAV/MnO_2_‐PEI NS Hybrid Complex

**Scheme 1:**
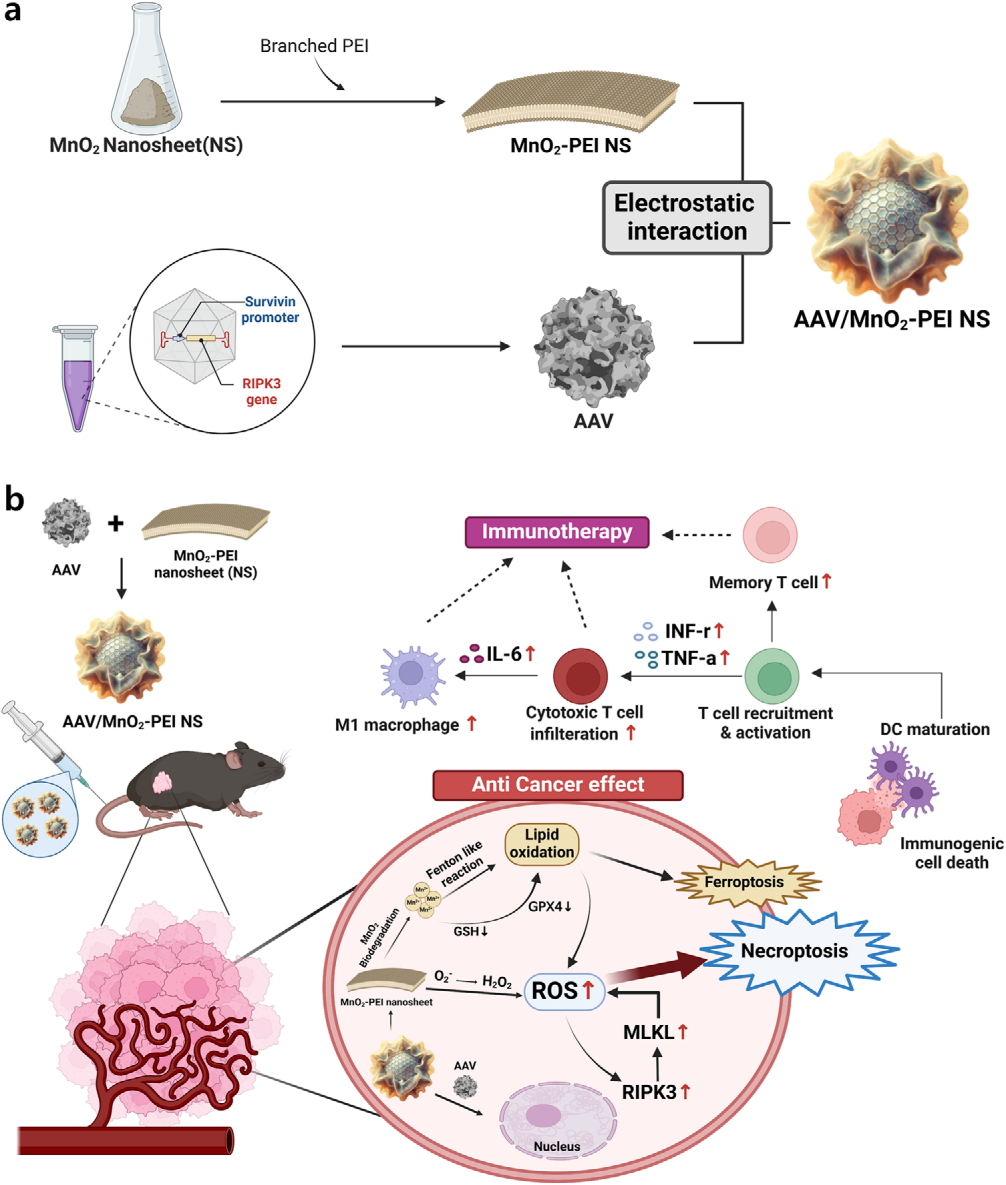
a) Schematic illustration of the preparation of MnO_2_‐PEI NS and AAV. b) Non‐apoptotic antitumor mechanism and immunotherapeutic mechanism of AAV/MnO_2_‐PEI NS nanoplatform. The figure was created with BioRender.com.

As illustrated in **Scheme** **1**a, the synthesis of multifunctional MnO_2_‐PEI nanosheet (NS) commenced with the reduction of potassium permanganate (KMnO_4_) to synthesize MnO_2_ in a 2D nanosheet morphology. These MnO_2_ NS were subsequently functionalized with 10 kDa branched PEI to impart a positively charged surface, enabling electrostatic complexation with the negatively charged AAV capsid. In this construct, MnO_2_ provides a functional scaffold with intrinsic ROS generation and glutathione (GSH) depletion capabilities, while the PEI modification enhances nanosheet stability, biocompatibility, and complexation efficiency. The successful integration of these components results in a robust platform for targeted gene delivery and enhanced therapeutic efficacy (Scheme 1b).

**Figure 1:**
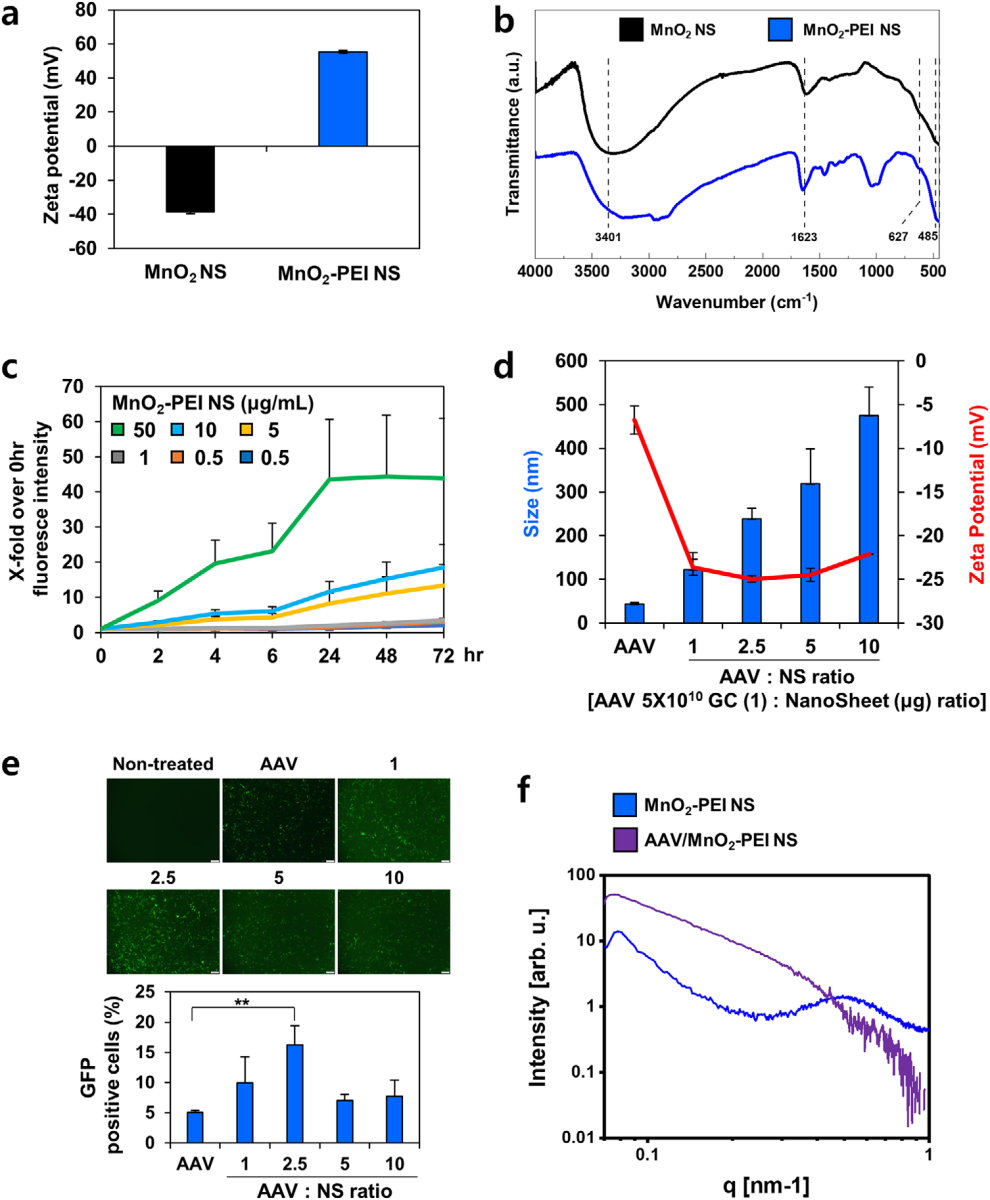
Characterization of MnO_2_‐PEI NS and AAV/MnO_2_‐PEI NS complex. a) Zeta‐potential profile of MnO_2_ NS and MnO_2_‐PEI NS. b) FTIR spectra of MnO_2_ NS and MnO_2_‐PEI NS. c) ROS production ability of MnO_2_‐PEI NS assessed by DCF intensity (*n* = 3). d) Hydrodynamic size and zeta potential of the AAV/MnO_2_‐PEI NS complex at various concentration of MnO_2_‐PEI NS (1–10 µg) and 5 × 10^10^ genome copy of AAV (*n* = 3). e) Fluorescence image of B16F10 cells transduced with GFP expressing AAV or AAV/MnO_2_‐PEI NS (1, 2.5, 5, and 10 AAV (gc): NS weight ratio). Original magnification: 100 x. Quantification of GFP‐positive cells by image J software (*n* = 3). f) SAXS analysis of MnO_2_‐PEI NS and AAV/MnO_2_‐PEI NS. Data are presented as mean ± SD; for panels (c–e). Statistical significances were assessed by one‐way ANOVA: ** *p* < 0.01.

Transmission electron microscopy (TEM) imaging confirmed that both pristine MnO_2_ NS and PEI‐functionalized MnO_2_ NS retained a distinct 2D sheet‐like morphology in ethanol solution (Figure S1, Supporting Information). The mean hydrodynamic size and distribution of MnO_2_ NS and MnO_2_‐PEI NS were assessed using dynamic light scattering (DLS). The average size of MnO_2_ NS was 282.9 nm, demonstrating a highly monodisperse characteristic with uniformly distributed 2D sheet‐like formations. Functionalization of MnO_2_ NS with PEI 10 kDa resulted in size to ≈282.8 nm (Figure S2, Supporting Information). Furthermore, zeta potential analysis demonstrated a marked shift from negative to strongly positive values upon PEI conjugation (**Figure** **1**a), thus confirming successful surface modification. Fourier transform infrared spectra (FT‐IR) pattern revealed that successful synthesis of MnO_2_ NS and MnO_2_‐PEI NS (Figure 1b). In order to assess the capacity for ROS‐generating, a DCFDA (2′‐7′‐Dichlorodihydrofluorescein diacetate) assay was employed (Figure 1c). A concentration‐dependent increase in fluorescence intensity validated the ROS‐generating functionality of MnO_2_‐PEI NS. While both MnO_2_ and MnO_2−_PEI NS displayed toxicity at higher concentrations, likely due to manganese ions (Mn^2+^)‐mediated oxidative stress, their use at lower concentrations mitigated nonspecific toxicity and enabled safer gene delivery (Figure S3, Supporting information).

To determine the optimal ratio for complexing AAV with MnO_2−_PEI NS, we progressively increased the amount of MnO_2−_PEI NS and evaluated key physicochemical parameters, including average size and surface charge, using DLS and zeta potential analysis. As shown in Figure 1d, the size of naked AAV was 43.7 ± 3.4 nm. When AAV was complexed at a genome copy (gc) to weight ratio with nanosheet of 2.5, the AAV/MnO_2−_PEI NS exhibited an average diameter of 238.4 ± 24.4 nm, indicating successful electrostatic interaction between the negatively charged AAV surface and the positively charged MnO_2−_PEI NS. Zeta potential analysis showed that the zeta potential shifted from negative to neutral charge as the amount of positively charged MnO_2_‐PEI nanosheets on the AAVs increased. This result suggests that in the presence of AAVs, the zeta potential is negatively charged, mainly due to the inherent negative charge of the AAVs, but the surface charge can be tuned by the introduction of MnO_2_‐PEI, allowing the MnO_2_‐PEI ratio to be optimized for high intracellular delivery while simultaneously optimizing the surface charge.

Subsequently, we assessed the transduction efficiency of these complexes by monitoring GFP expression at various AAV (gc) to MnO_2−_PEI NS (weight) ratios (Figure 1e). Complexes containing MnO_2−_PEI NS displayed higher GFP expression compared to AAV alone, with peak efficiency observed at a ratio of 2.5. This enhanced delivery has been attributed to the release of Mn^2+^ ion from the nanosheets, which have been demonstrated to promote AAV‐mediated gene expression.^[^ ^22^ ^]^ However, further increases in the gc by weight with nanosheet ratios (5 and 10) were shown to decrease GFP expression, suggesting that maintaining an appropriate ratio of AAV to nanosheets is necessary to increase gene expression efficiency. Moreover, gc to weight ratio of 2.5 showed 68% encapsulation rate (Figure S4, Supporting Information). Further structural characterization of MnO_2_‐PEI NS and its complexes with AAV was performed based on the SAXS analysis of AAV (Figure S5, Supporting Information). As demonstrated in Figure 1f, the results of SAXS scans for MnO_2_‐PEI NS and AAV/MnO_2_‐PEI NS complexes at a gc to weight ratio of 2.5 are presented. Guinier analysis revealed that the radius of gyration (R_g_) of the complex is 13.3 nm. In a spherical shell model, the diameter of complex was calculated as 30.8 nm, which supports the nanosheet‐wrapped morphology. Notably, the broad form factor at *q* = 0.5 nm^−1^ observed in the MnO_2−_PEI NS sample is not present in the AAV/MnO_2−_PEI NS complex, suggesting that most of MnO_2−_PEI NS effectively wraps the AAV at a gc to weight ratio of 2.5. Consequently, a nanosheet ratio of 2.5 gc to weight was determined to be the optimal condition for AAV/MnO_2_‐PEI NS composites.

### 2.2 Non‐Apoptotic Cancer Cell Death Induced by AAV/MnO_2_‐PEI NS

**Figure 2:**
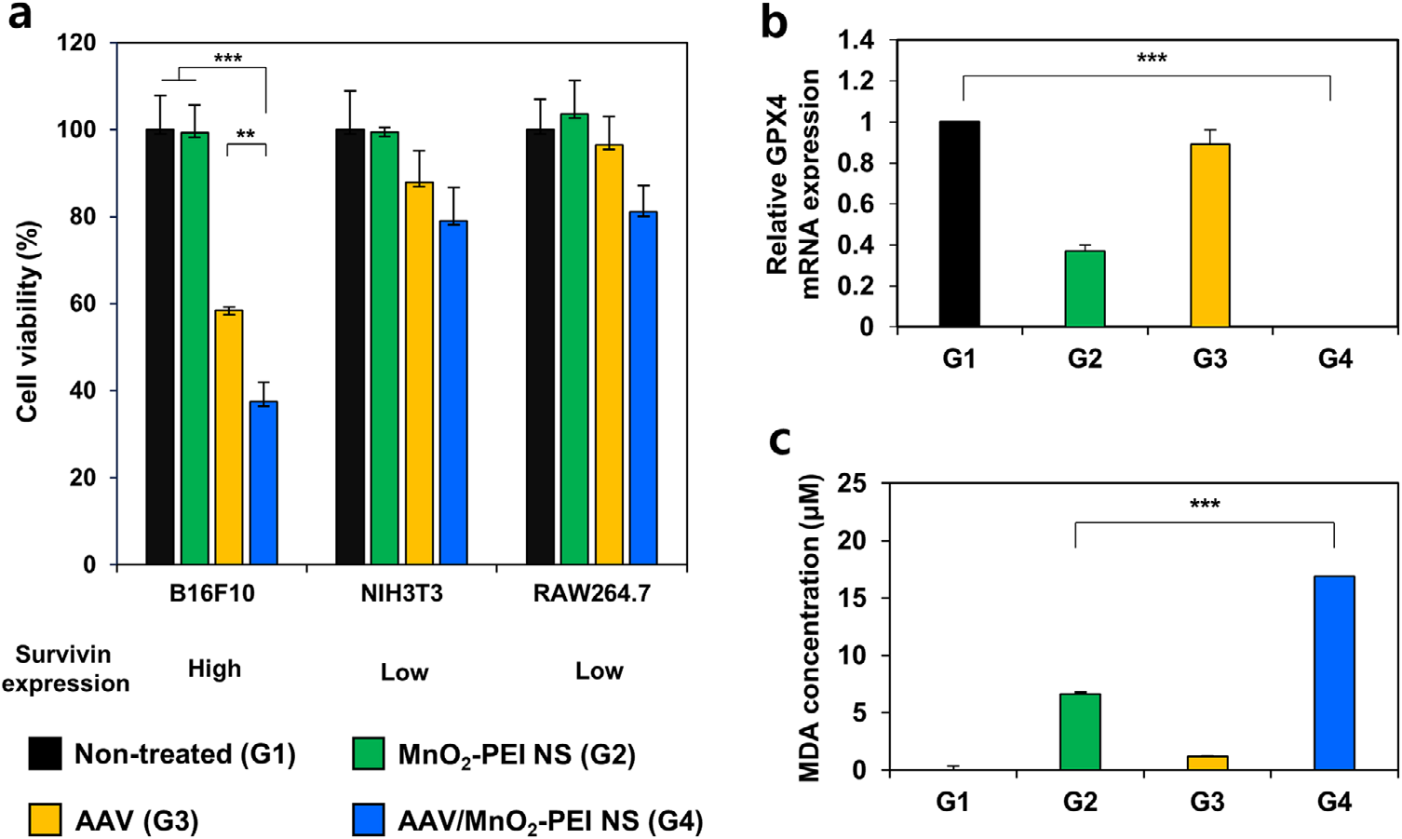
Non‐apoptotic cancer cell death induced by AAV/MnO_2_‐PEI NS. a) Cell viability of B16F10, NIH3T3, and RAW264.7 cells after treatment with different formulations for 72 h (*n* = 3). b) GPX4 mRNA expression after treatment with different formulations for 72 h (*n* = 3). c) Quantification of MDA level in cells after treatment with different formulation for 72 h (*n* = 3). Data are presented as mean ± SD. Statistical significances were assessed by one‐way ANOVA: ** *p* < 0.01, *** *p* < 0.001.

To selectively induce necroptosis in tumor cells, an AAV vector was constructed that encoded RIPK3, a central regulator of necroptotic cell death. This vector was constructed under the control of the tumor‐specific survivin promoter, which restricts transgene expression predominantly to malignant cells, thereby minimizing off‐target effects on normal tissues.^[^ ^23^, ^24^ ^]^ Cell viability assays conducted on three distinct murine cell lines – B16F10 melanoma (high survivin expression), NIH3T3 fibroblasts, and RAW264.7 macrophages (both with low survivin expression) – confirmed the selectivity of the platform (**Figure** **2**a). Survivin expression levels in these cell lines were confirmed by qRT‐PCR (Figure S6, Supporting Information). Notably, in the B16F10 cells, treatment with the AAV/MnO_2−_PEI NS complex (G4) resulted in significant level of cell death in comparison to untreated controls (G1), MnO_2−_PEI alone (G2), and AAV alone (G3). It is important to note that G3 and G4 treatments produced negligible cell killing effects in the NIH3T3 or RAW264.7 cells, confirming the tumor‐specific effect of the survivin promoter–driven selective expression of RIPK3. To validate the detailed mechanism underlying the selective cancer cell killing induced by survivin‐mediated transgene expression, we performed Western blot analysis to confirm the expression of RIPK3 and MLKL, which is a key signaling molecule in necroptosis. As shown in Figure S7 (Supporting Information), we confirmed that the highest expression levels of RIPK3 and MLKL were observed in the AAV/MnO_2−_PEI NS–treated group (G4), indicating robust activation of the necroptotic pathway. To further determine whether the stimulation of necroptosis induces immunogenic cell death (ICD), we evaluated the expression of calreticulin (CRT) as a key marker. Immunocytochemical (ICC) staining for CRT showed a markedly increased signal in the AAV/MnO_2−_PEI group (G4) compared with all other groups (Figure S8, Supporting Information). This suggests that the AAV/MnO_2−_PEI complex not only triggers necroptotic pathways via RIPK3 but also activates ICD, potentially enhancing antitumor immunity.

Following AAV/MnO_2−_PEI NS internalization, MnO_2−_PEI nanosheets are biodegraded to Mn^2^⁺, which in turn drives a fenton‐like reaction and depleting intracellular GSH. As a result, GPX4 expression is diminished, and FPN (ferroportin) levels increase, promoting lipid peroxidation and thus ferroptosis.^[^ ^25^ ^]^ Indeed, Quantitative reverse transcription PCR analysis (Figure 2b) revealed that among all the groups, the AAV/MnO_2−_PEI NS–treated group (G4) exhibited the lowest GPX4 mRNA levels, suggesting robust induction of ferroptosis. Additionally, FPN mRNA levels were significantly elevated, likely due to the release of Mn^2^⁺ from the nanosheets (Figure S9, Supporting Information). To assess lipid peroxidation, we quantified malondialdehyde (MDA), a well‐established marker of lipid peroxides. As shown in Figure 2c, The AAV/MnO_2_‐PEI‐treated group exhibited the highest MDA concentration, confirming that GPX4 downregulation triggered lipid oxidation and providing induction of ferroptosis. In conjunction with the elevated necroptosis markers (Figure S7, Supporting Information), these findings imply that the necroptotic pathway (mediated by RIPK3/MLKL) and the ferroptosis pathway (Figure 2b,c) work synergistically to induce potent cancer cell killing (Figure 2a). Furthermore, the pronounced ICD signal, as evidenced by the upregulation of CRT (Figure S8, Supporting Information), underscores the multifaceted mechanism underlying the efficacy of the AAV/MnO_2−_PEI NS complex. Taken together, these results strongly support that the combination of necroptosis and ferroptosis orchestrates an effective non‐apoptotic cancer cell killing strategy, ultimately affirming the therapeutic potential of this platform.

### 2.3 Biocompatibility and Bio‐Distribution of AAV/MnO_2_‐PEI NS

**Figure 3:**
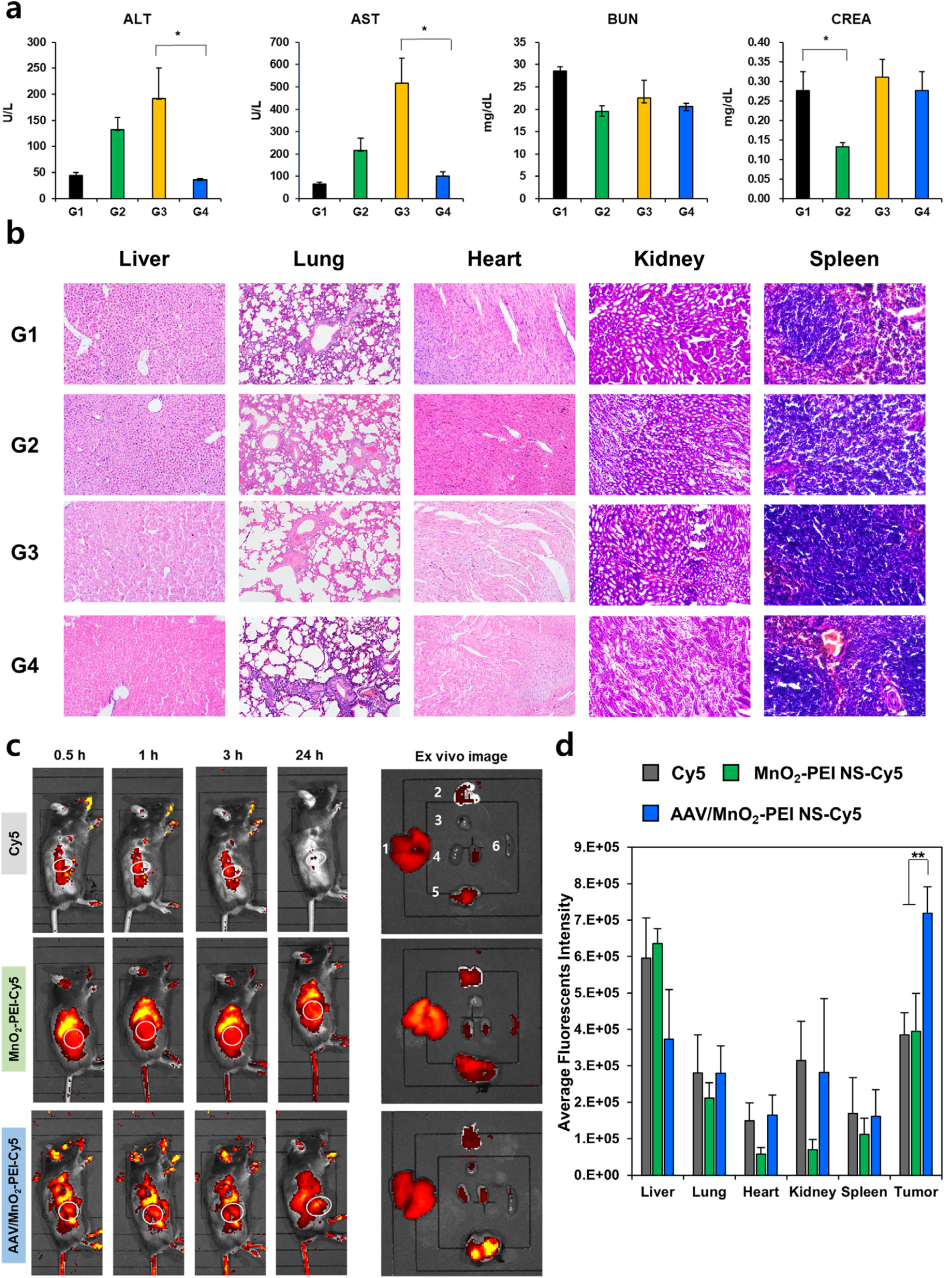
Biodistribution and biocompatibility of AAV/MnO_2_‐PEI NS in vivo. a) biochemical parameters of liver function and kidney function profile (*n* = 3). b) H&E staining of major organs in each group. c) Fluorescence distribution of Cy5‐labeled MnO_2_‐PEI and AAV/MnO_2_‐PEI in mice. d) Fluorescence images of isolated organs 24 h after administration (1: Liver, 2: Lung, 3: Heart, 4: Kidney, 5: Tumor, 6: Spleen) and quantitative measurement of fluorescence intensity in isolated organs following 24 h after administration (*n* = 3). Data are presented as mean ± SD; for panels (a,d). Statistical significances were assessed by one‐way ANOVA: * *p* < 0.05, ** *p* < 0.01.

It is essential that biocompatibility is a prerequisite for translational application, and a thorough evaluation of systemic safety must precede in vivo therapeutic evaluation. Despite the extensive utilization of AAV vectors for gene delivery, their systemic administration is frequently constrained by immunogenicity, rapid hepatic clearance, and associated toxicity.^[^ ^26^ ^]^ Host immune responses to the AAV capsid can trigger adverse inflammatory reactions, compromising both safety and therapeutic efficacy. To investigate whether MnO_2_ in AAV‐MnO_2−_PEI NS could act as a shield to mitigate AAV‐induced hepatotoxicity, serum levels of alanine aminotransferase (ALT) and aspartate aminotransferase (AST) were quantified following systemic administration. As demonstrated in **Figure** **3**a, mice treated with naked AAV (G3) exhibited elevated ALT and AST levels in comparison to the PBS control (G1), indicative of hepatic stress. In contrast, the AAV/MnO_2−_PEI NS group (G4) demonstrated ALT and AST levels that were comparable to the control, thereby suggesting that nanosheet complexation with MnO_2−_PEI effectively mitigates AAV‐induced liver toxicity. Further blood chemistry analysis revealed no significant changes in blood urea nitrogen (BUN) or creatinine (CREA), providing further evidence of renal safety. Histopathological analysis further supported these findings. Hematoxylin and eosin (H&E) section staining of the major organs revealed no notable abnormal pathological manifestations in any group (Figure 3b).

To evaluate the biodistribution of the AAV/MnO_2−_PEI NS complex, we performed intravenous injection of Cy5‐labeled formulations, including Cy5 dye alone, MnO_2_‐PEI NS‐Cy5, and AAV/MnO_2_‐PEI NS‐Cy5. As shown in Figure 3c, time‐course imaging revealed that AAV/MnO_2−_PEI NS‐Cy5 exhibited significantly reduced liver accumulation and enhanced tumor localization compared to free Cy5. This observation was further corroborated by ex vivo imaging and the quantification of Cy5 intensity in each excised organ (Figure 3d). The favorable biodistribution profile of AAV/MnO_2−_PEI NS is likely due to its negatively charged surface and optimal nanoscale dimensions, both of which facilitate enhanced tumor accumulation through the improved permeability and retention (EPR) effect.^[^ ^27^ ^]^ Collectively, the data obtained highlight the ability of MnO_2_‐PEI nanosheets to improve the safety and tumor accumulation efficiency of systemically delivered AAV vectors. The dual benefit of reduced off‐target toxicity and improved tumor accumulation supports the suitability of AAV/MnO_2_‐PEI NS as a promising platform for in vivo cancer gene therapy.

### 2.4 Anti‐Tumor Efficacy of AAV/MnO_2_‐PEI NS

**Figure 4:**
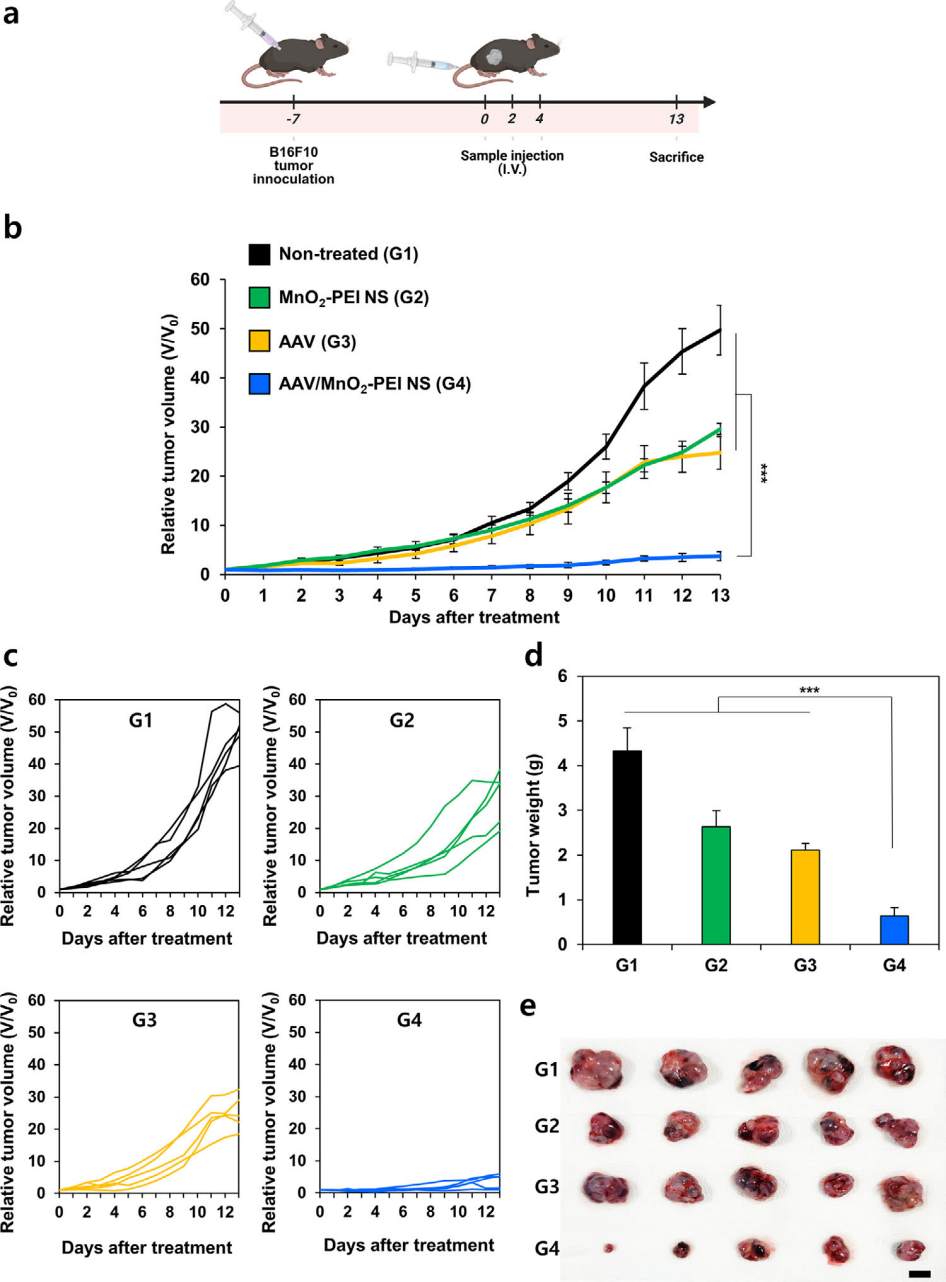
Antitumor efficacy of AAV/MnO_2_‐PEI NS in vivo. a) Scheme illustration of AAV/MnO_2_‐PEI treatment process and animal experimental design on B16F10 tumor‐bearing mice. b) Tumor growth inhibition curves of B16F10 tumor‐bearing mice subjected to different treatment (*n* = 5). c) Individual tumor volume change curves of B16F10 tumor‐bearing mice following different treatment. d) The tumor weights following different treatment after 13 days. e) Tumor photographs of dissected tumors after 13 days. Data are presented as mean ± SD; for panels (b,d). Statistical significances were assessed by one‐way ANOVA: *** *p* < 0.001.

To evaluate the antitumor efficacy of AAV/MnO_2_‐PEI NS in vivo, B16F10 tumor‐bearing mice were randomly assigned to four groups: PBS (G1), MnO_2_‐PEI NS (G2), AAV (G3), and AAV/MnO_2_‐PEI NS (G4). All groups received three intravenous injections at two‐day intervals (**Figure** **4**a). Tumor volumes and body weights of all groups were monitored daily for 13 days. As shown in Figure 4b,c G1, G2, and G3 exhibited limited tumor suppression, whereas G4 demonstrated the most pronounced antitumor effect with significantly reduced tumor volume. Furthermore, the body weight of treated mice remained stable throughout the study, indicating the biosafety of AAV/MnO_2−_PEI NS (Figure S10, Supporting Information). At day 13, the mice were euthanized and tumors were excised, weighed, and photographed. As shown in Figure 4d,e, the weight of the tumors showed the following order: G1> G2> G3> G4, underscoring the superior therapeutic efficacy achieved by combining AAV with MnO_2−_PEI NS.

In elucidate the underlying mechanism of tumor suppression, the expression levels of therapeutic transgene (RIPK3) were analyzed in tumor tissues at 3 days after the administration of the last treatment. RIPK3 was robustly expressed only in the G4 (AAV/MnO_2−_PEI NS) tumors, whereas no detectable signal was observed in the PBS, MnO_2−_PEI NS, or AAV groups, indicating successful tumor‐targeted gene delivery and expression mediated by the MnO_2−_PEI NS (Figure S11, Supporting Information). The results of the histological evaluation provided further mechanistic insight. The findings on cell death and cell proliferation inhibition resulting from AAV/MnO_2_‐PEI NS treatment were verified by H&E, Ki‐67, and TdT‐mediated dUTP nick‐end labeling (TUNEL) staining (Figure S12, Supporting Information). H&E staining revealed extensive nuclear fragmentation and nucleolysis in the G4 tumor sections, indicative of significant tumor cell destruction. In contrast, G2 and G3 showed comparatively less necrosis, and the G1 tumor remained largely intact. Immunohistochemical staining also supported these observations: TUNEL assays detected markedly higher levels of apoptotic cells in G4, whereas Ki‐67 staining indicated reduced proliferation relative to other treatment groups. Together, these data point to a strong therapeutic synergy between RIPK3 gene delivery and MnO_2−_PEI NS–mediated enhancement of cell death pathways. From a broader perspective, these findings suggest that AAV/MnO_2−_PEI NS has the potential to overcome common limitations of systemic gene delivery by improving tumor specificity and reducing off‐target effects, as evidenced by the lack of significant weight loss. Furthermore, the capacity to effectively express a potent transgene such as RIPK3 within the tumor microenvironment has the potential to enhance other cell death mechanisms (e.g., necroptosis or ferroptosis) and potentially augment immunogenic responses. Although further investigation is necessary to delineate the long‐term safety and molecular mechanisms of this combination strategy, the present results underscore its promise as a potent and safe therapeutic modality for malignant melanoma and potentially other solid tumors.

### 2.5 Antitumor Immune Response Boosted by AAV/MnO_2_‐PEI NS

**Figure 5:**
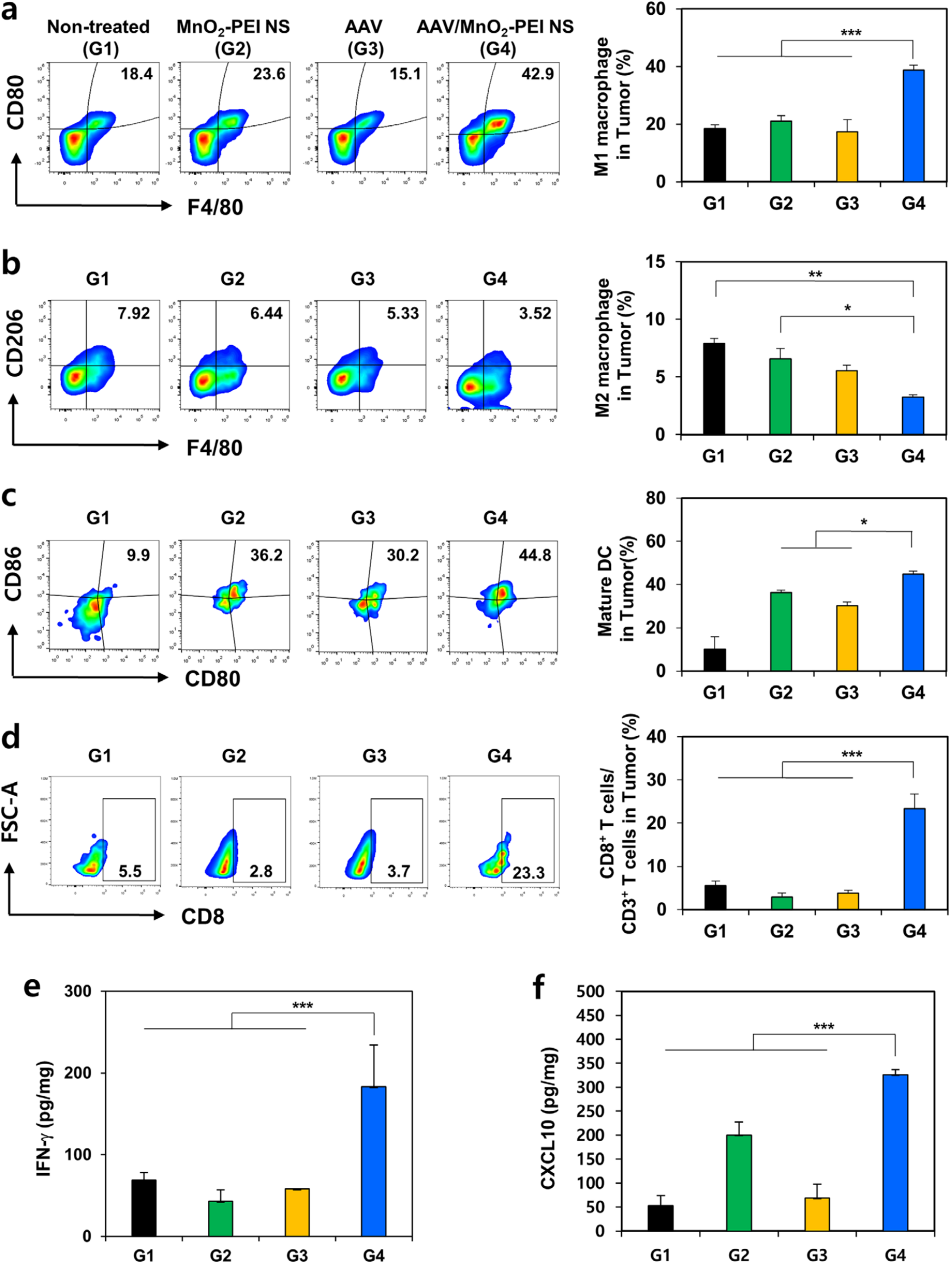
Antitumor immune response boosted by AAV/MnO_2_‐PEI NS in vivo. a) Representative flow cytometry analysis plots and quantification analysis of M1 macrophage (CD45^+^CD11b^+^F4/80^+^CD80^+^) in tumor (*n* = 3). b) Representative flow cytometry analysis plots and quantification analysis of M2 macrophage (CD45^+^CD11b^+^F4/80^+^CD206^+^) in tumor (*n* = 3). c) Representative flow cytometry analysis plots and quantification analysis of matured DCs (CD45^+^CD11c^+^CD80^+^CD86^+^) in tumor (*n* = 3). d) Representative flow cytometry analysis plots and quantification analysis of CD8^+^ T cells (CD3^+^CD8^+^) in tumor (*n* = 3). Cytokine levels of (e) INF‐γ and (f) CXCL10 in tumor (*n* = 3). Data are presented as mean ± SD. Statistical significances were assessed by one‐way ANOVA: * *p* < 0.05, ** *p* < 0.01, *** *p* < 0.001.

ROS and elevated O_2_ levels, and Mn^2+^ can repolarize tumor‐associated macrophages from an immunosuppressive M2 phenotype toward a pro‐inflammatory M1 state. This, in turn, has been demonstrated to remodel the TME in a manner conducive to antitumor immunity.^[^ ^28^, ^29^, ^30^ ^]^ Based on this we hypothesized that co‐delivery of a necroptosis‐inducing gene (RIPK3) with MnO_2_–PEI NS would synergistically enhance immune‐mediated tumor suppression. RIPK3 expression promotes necroptosis, releasing DAMPs that facilitate dendritic cell (DC) maturation and T‐cell recruitment. Simultaneously, Mn^2^⁺, which is generated via the biodegradation of MnO_2−_PEI NS, can induce ferroptosis, increase ROS production and trigger ICD, further activating immune cells within the TME. These mechanisms collectively convert a “cold” tumor, characterized by poor immune infiltration, into a “hot” tumor enriched with effector immune cells. To evaluate the immunomodulatory capacity of this platform in vivo, the study assessed the polarization of macrophages, the maturation of dendritic cells (DCs), and the infiltration of CD8^+^ T‐cells following treatment with AAV/MnO_2_–PEI NS. Subsequent flow cytometry analysis revealed that the G4 group (AAV/MnO_2−_PEI NS) exhibited a significantly increased proportion of M1 macrophages (CD80⁺F4/80⁺) and a concomitant reduction in M2 macrophages (CD206⁺F4/80⁺) compared to G1–G3 (**Figure** **5**a,b). These results suggest that ROS and Mn^2+^ derived from the nanosheets play a key role in reshaping the immunosuppressive TME into one that favors immune activation. Concurrently, the maturation of dendritic cells (DCs) was significantly augmented in the G4 group, as demonstrated by the upregulation of co‐stimulatory molecules CD80 and CD86 (Figure 5c). This effect is likely driven by increased DAMP release from RIPK3‐mediated necroptosis and Mn^2+^‐amplified ICD. Consequently, the number of infiltrating CD8^+^ T cells was also significantly elevated in the G4 group (Figure 5d), highlighting the downstream activation of cytotoxic T‐cell responses. The findings demonstrate a synergistic mechanism in which the dual action of necroptosis and ferroptosis not only promotes tumor cell death but also potentiates adaptive immunity. To further validate the immunological transition from a “cold” to a “hot” tumor microenvironment, we measured the levels of key cytokines associated with Th1‐type immune responses. As shown in Figure 5e,f, the AAV/MnO_2−_PEI NS group (G4) exhibited the highest concentrations of IFN‐γ and CXCL10 within the tumor tissue. These findings are consistent with the enhanced DC maturation observed in Figure 5c and the increased CD8⁺ T‐cell infiltration shown in Figure 5d. Increased IFN‐γ is indicative of robust T‐cell activation and a pro‐inflammatory milieu, whereas CXCL10 has been implicated in the recruitment and activation of T cells, thereby augmenting Th1‐driven immune responses. Collectively, these results suggest that AAV/MnO_2−_PEI NS mediates a comprehensive immune reprogramming within the tumor, characterized by the polarization of macrophages toward a tumoricidal M1 phenotype, the maturation of DCs, and the increased infiltration of CD8⁺ T cells. This immunological environment is further boosted by high levels of Th1‐associated cytokines, supporting an overall shift toward a more immunogenic, “hot” tumor phenotype. Consequently, the combination of RIPK3 expression and MnO_2−_PEI nanosheets represents a potent strategy for enhancing antitumor immunity and may hold promise for integration with other immunotherapeutic modalities.

### 2.6 AAV/MnO_2_‐PEI NS‐Mediated Prevention of Post‐Operative Tumor Recurrence

**Figure 6:**
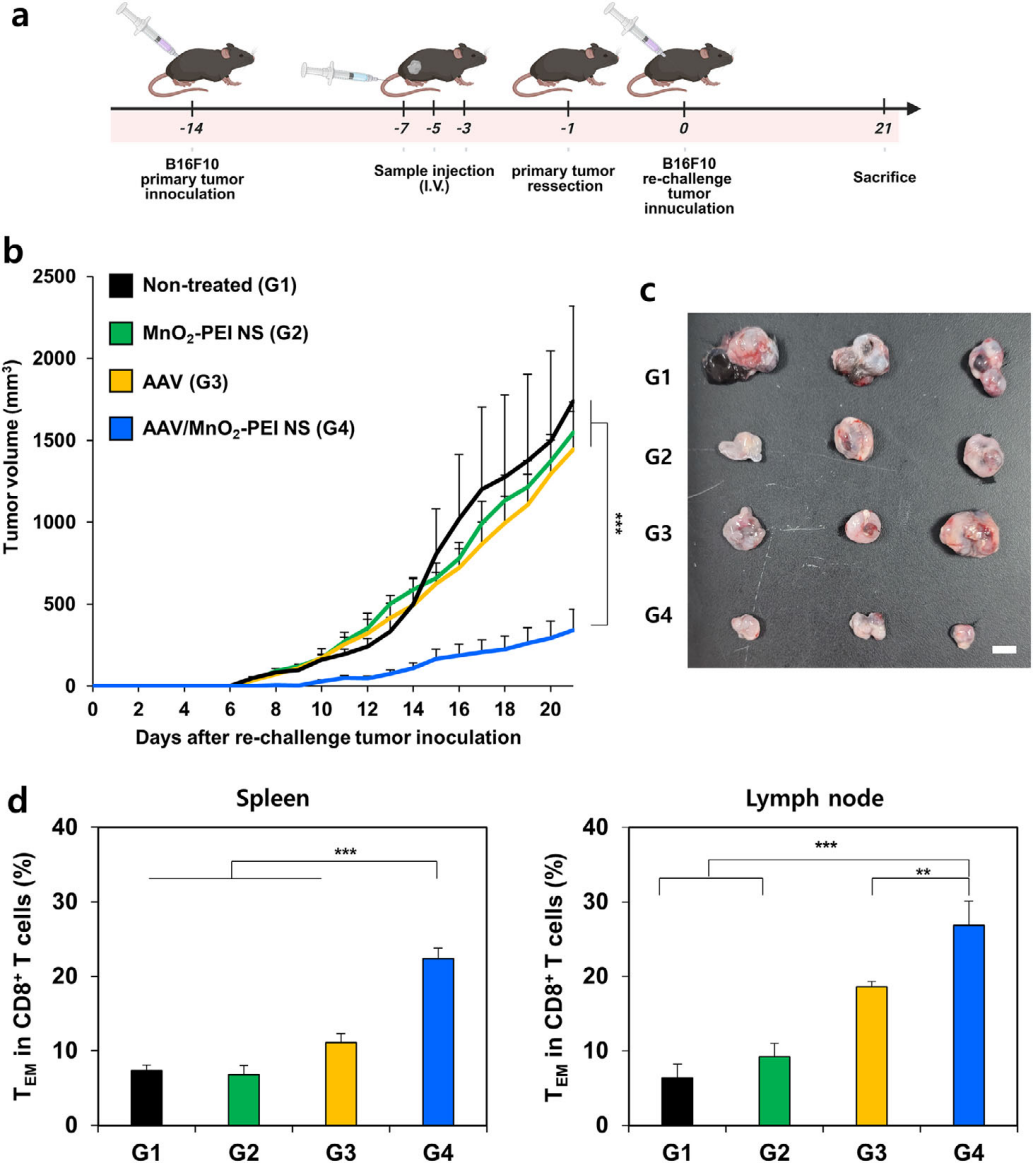
Inhibition of post‐surgery tumor recurrence by AAV/MnO_2_‐PEI NS in vivo. a) Scheme illustration of AAV/MnO_2_‐PEI NS treatment process and animal experimental design in tumor recurrence model. b) Tumor growth inhibition curves of re‐challenged tumors in various treatment (*n* = 3). c) Tumor photographs of dissected tumors at day 21 after re‐challenge tumor inoculation. d) Quantification of CD8^+^ effector memory T cells (T_EM_ cells, CD3^+^CD8^+^CD44^+^CD62L^−^) in spleen and lymph node on day 21 after re‐challenge tumor inoculation. Data are presented as mean ± SD; for panels (b,d). Statistical significances were assessed by one‐way ANOVA: ** *p* < 0.01, *** *p* < 0.001.

The antitumor efficacy and robust immune activation observed in Figures 4 and 5 were the basis of further investigation into whether AAV/MnO_2_‐PEI NS could prevent tumor recurrence. As shown in **Figure** **6**a, B16F10 cells were subcutaneously injected into the right flank of mice and treated three times according to the established regimen. After 2 days of final administration, the primary tumors were surgically removed, and the mice were re‐challenged with B16F10 cells in the contralateral flank. It is noteworthy that while the growth of tumor in the re‐challenged groups (G1–G3) progressed with only minimal inhibition, the AAV/MnO_2−_PEI–treated group (G4) exhibited significant suppression of tumor outgrowth despite the absence of additional treatment (Figure 6b). Measurement of the extracted tumors weight revealed that the G4 group had the most pronounced impact, which was further confirmed by photographic evidence (Figure 6c; Figure S13, Supporting Information). Memory T cells are widely recognized for their pivotal contribution in sustaining antitumor immune responses and preventing tumor recurrence.^[^ ^31^, ^32^ ^]^ To further investigate the underlying immune mechanisms, tumors were harvested at day 21 and assessed the population of effector memory T (T_EM_) cells in the spleen and lymph nodes was assessed (Figure 6d). The result of flow cytometric analysis revealed that AAV/MnO_2−_PEI–treated group (G4) exhibited substantially higher T_EM_ levels compared with other groups, suggesting that AAV/MnO_2−_PEI NS elicited a durable, systemic immune response. Collectively, these results emphasize the capacity of AAV/MnO_2_‐PEI NS to not only eradicate primary tumors but also to prevent recurrence through the induction of durable, memory‐driven antitumor immunity. The establishment of long‐lasting immune protection following a short treatment course highlights the translational potential of this strategy as an adjuvant or standalone immunotherapeutic approach for the management of post‐surgical tumor recurrence.

## 3 Conclusion

In conclusion, the results demonstrate that combination of AAV‐mediated RIPK3 expression with MnO_2−_PEI nanosheets elicits robust and multifaceted antitumor activity. Mechanistically, this novel gene delivery platform leverages necroptosis and ICD, driving the release of DAMPs and tumor antigens that mature DCs and recruit cytotoxic T cells. Concurrently, the generation of ROS by manganese, in conjunction with ferroptosis and RIPK3 expression‐induced necroptosis, further modifies the immunosuppressive tumor microenvironment, thereby promoting M1 macrophage polarization and favoring a Th1‐type immune response. The systemic administration of AAV/MnO_2−_PEI NS not only alleviates the liver tropism commonly associated with naked AAV but also achieves elevated tumor accumulation and minimal off‐target toxicity. Critically, a tumor re‐challenge model revealed durable protection in mice treated with the AAV/MnO_2−_PEI NS complex, evidenced by high levels of effector memory T cells, increased intratumoral and serum IFN‐γ, and significant suppression of tumor recurrence. Collectively, these findings highlight the potential of AAV/MnO_2−_PEI NS as a versatile and potent platform for cancer gene immunotherapy, offering a promising approach to convert immunologically cold tumors into hot tumors and providing a strong foundation for future clinical translation. Furthermore, its suitability for the combined delivery also suggests its potential for transporting various functional cargoes, including therapeutic genes and gene‐editing materials. This could lead to its use in a wider range of applications in genetic and regenerative medicine.

## Conflict of Interest

The authors declare no conflict of interest.

## Supporting information



Supporting Information

## Data Availability

The data that support the findings of this study are available from the corresponding author upon reasonable request.
