# Supplementary material for: Nanoshield‐Assisted Viral Gene Therapy with Induction of Non‐Apoptotic Cell Death and Durable Antitumor Immunity
Source: Adv Sci (Weinh). 2025 Aug 4;12(41):e07550. doi: 10.1002/advs.202507550 (PMC12591101; doi:10.1002/advs.202507550)
Supplement: Supplementary file 1 — Supporting Information [file ADVS-12-e07550-s001.docx]

**Supporting Information**

**Nanoshield-Assisted Viral Gene Therapy with Induction Non-Apoptotic Cell Death and Durable Antitumor Immunity**

Soo-Hwan Lee*^a^*, Yunkyeong Cho*^a^*, Seunghwan Bang*^b,c^*, Daeun Sung*^d^*, Jahyun Koo*^d,e^*, Seoyoung Kim*^a^*, Youngil Koh*^f^*, Hojun Kim*^b,c^*, Hyojin Lee*^a,b,g,∗^*

*^a^Biomaterials Research Center, Korea Institute of Science and Technology (KIST), Seoul, 02792, Republic of Korea*

*^b^Division of Biomedical Science and Technology, KIST School, Korea University of Science and Technology, Seoul, 02792, Republic of Korea*

*^c^Center for Advanced Biomolecular Recognition, Korea Institute of Science and Technology (KIST), Seoul 02792, Republic of Korea*

*^d^School of Biomedical Engineering, Korea University, Seoul, 02841, Republic of Korea*

*^e^Interdisciplinary Program in Precision Public Health, Korea University, Seoul 02841, Republic of Korea*

*^f^Department of Internal Medicine, Seoul National University Hospital, Seoul, 03080, Republic of Korea*

*^g^SKKU-KIST, Department of Integrative Biotechnology, College of Biotechnology and Bioengineering, Sungkyunkwan University, Suwon, Gyeonggi, 16419, Republic of Korea.*

**Corresponding authors.*

**Email: hyojinlee@kist.re.kr*

**Experimental Section**

## **Materials**

SDS (sodium dodecyl sulfate) solution, Molecular Biology Grade (10% w/v) was purchased from Promega (Madison, WI). Sulfuric acid (H_2_SO_4_) was purchased from Daejung chemicals (Seoul, Korea). Potassium permanganate (KMnO_4_), deoxyribonuclease I, and type IV collagenase were purchased from Sigma-Aldrich (St. Louis, MO). Polyethyleneimine (PEI), Branched, MW 10,000(PEI 10K) and PEI, Linear, MW 25,000, transfection grade (PEI 25K™) was purchased from Polysciences (Warrington, PA). SnakeSkin Dialysis Tubing, fetal bovine serum (FBS), Hanks' Balanced Salt Solution (HBSS), TRIzol Reagent, Pierce™ BCA Protein Assay Kits, and ProLong™ Gold Antifade Mountant with DNA Stain DAPI was purchased from Thermo Fisher Scientific (Waltham, MA). The Primers were purchased from Cosmogenetech (Seoul, Korea). Anti-Calreticulin, Anti-MLKL, Anti-RIPK3, Anti-Phospho-MLKL, Anti-Phospho-RIPK3 antibody were purchased from Cell Signaling Technology (Beverly, MA). Anti-GAPDH, Anti-Ki67, Anti-Rabbit IgG H&L Alexa Fluor 488, Anti-Rabbit IgG H&L (HRP) antibody, H&E staining kit, TUNEL assay (BrdU-Red) kit, and DCFDA/H2DCFDA cellular ROS assay kit were purchased from Abcam (Cambridge, UK). Anti-CD80-BV605, Anti-CD86-BV785, Anti-CD3-BV510, Anti-CD62L-PE/Cyanine7, Anti-CD44L-PE/Cyanine5, Anti-CD8a-BV711, Anti-CD11c-PE/Cyanine7, Anti-CD11b-PerCP/Cyanine5.5, Anti-CD45-Alexa Fluor 700, Anti-F4/80-APC, Purified Anti-CD16/32 antibody were purchased from Biolegend (San Diego, CA). Mouse IFN-γ ELISA assay kit (DY485-05) and mouse CXCL-10 ELISA assay kit (DY-466-05) were purchased from R&D systems (Minneapolis, Minn). Dulbecco's modified Eagle's medium (DMEM), Penicillin/streptomycin (P/S), and Phosphate-buffered saline (PBS, 10×) were purchased from WELGENE (Daegu, Korea). pAAV-CMV-GFP (plasmid #67634), pAAV2/2 (plasmid #104963), pAdDeltaF6 (plasmid #112867) were purchased from Addgene (Cambridge, MA). pAAV-SURVIVIN-RIPK3 was purchased from Vector builder (Shanghai, China). Bovine serum albumin (BSA), RIPA Cell Lysis Buffer, Xpert Duo Inhibitor Cocktail Solution, Laemmli Sample Buffer were purchased from GenDEPOT (Katy, TX). Maxime RT PreMix Kit was purchased from iNtRON Biotechnology (Seoul, Korea). Ez-Lipid peroxidation (TBARS) assay kit was purchased from DogenBio (Seoul, Korea).

## **Instrumental methods**

Transmission electron microscope (TEM) images were obtained by JEM-1001 (JEOL Ltd., Tokyo, Japan). Hydrodynamic size and zeta potential of nanoparticles were measured by a Zetasizer NS90 (Malvern Panalytical Ltd, Malvern, UK). FT-IR spectra were recorded by Nicolet iS50 spectrophotometer (Thermo Fisher Scientific). Absorbance for ELISA and Luminescent of CellTiter-Glo® were determined by Promega’s GloMax Discover System (Promega). Confocal laser scanning microscope (CLSM) image was monitored with Olympus FV-3300 (Olympus, Tokyo, Japan). Western Blot image was visualized using the iBright 750 Imaging System (Thermo Fisher Scientific). Reverse transcription-quantitative real time PCR and quantitative real time PCR were performed using QuantStudio 1 real-time PCR detection system (Thermo Fisher Scientific). In vivo biodistribution fluorescence images were obtained by real-time optical imaging system IVIS Lumina K (PerkinElmer, Waltham, MA). Flow cytometry data were measured by MA900 (SONY, Tokyo, Japan) and analyzed using Flow Jo™ software (BD Biosciences, Franklin Lakes, NJ). Quantitative analysis was conducted using ImageJ software version 1.50b (National Institutes of Health, Bethesda, MD)

## **Synthesis of MnO_2_-PEI Nanosheet (NS)**

The MnO_2_ NS were synthesized according to the previously reported method^[1]^. SDS solution (0.2 M, 32 mL) and H_2_SO_4_ solution (0.1 M, 1.6 mL) were added into 283.2 mL distilled water and heated at 95℃ for 15 min. KMnO_4_ solution (0.05 M, 3.2 mL) was rapidly added into SDS solution to start the reaction. The reaction mixture was heated for 60 min and allowed to cool to room temperature. The synthesized MnO_2_ nanosheets were purified by centrifugation and were dispersed in distilled water. Then, MnO_2_ NS were added to the PEI 10K solution at a mass ratio of MnO_2_: PEI 10K = 1:1.5. The reaction mixture was sonicated for two hours. Finally, the synthesized MnO_2_ NS coating PEI 10K was dialyzed using membrane tube for purification.

## **Cell lines and animal models**

AAVpro 293T cells were purchased from Takara Bio (Tokyo, Japan). B16F10 cells, NIH3T3 cells, RAW264.7 cells were all purchased from the American Type Culture Collection. All cell lines were culture in DMEM medium with 10% FBS and 1% penicillin-streptomycin. C57BL/6 mice (male, 6 weeks old) were provided from Nara biotech (Seoul, Korea). All animal experiment protocols were performed according to the laboratory animal use protocol approved by the Institutional Animal Care and Use Committee (IACUC) of Korea Institute of Science and Technology (KIST) (Approval ID: KIST-IACUC-2024-071).

## **AAV production, purification and titration**

AAV2-CMV-GFP and AAV2-Survivin-RIP3 transgene viruses were produced as previously described using chloroform extraction (Davidsson et al., 2020; Negrini et al., 2020). Briefly, AAVpro 293T cells were triple transfected with ITR-transgene, pAAV2/2 and the helper plasmid AdDeltaF6 using PEI25K^TM^. AAVs were harvested 72 h post-transfection using polyethylene glycol 8000 (PEG8000) precipitation and chloroform extraction followed by PBS exchange in concentration columns. Purified AAV viruses were tittered using a quantitative PCR-based method, with primers specific for the ITRs (forward primer 5′-CGG CCT CAG TGA GCGA-3′ and reverse primer 5′-GGA ACC CCT AGT GAT GGA GTT-3′).

## **Characterization of MnO_2_ NS and MnO_2_-PEI NS**

TEM image was used to characterize the morphology of MnO_2_ NS and MnO_2_-PEI NS. Size and zeta potential were analyzed by Zetasizer NS90. Additionally, Fourier-transform infrared (FT-IR) spectroscopy was performed to analyze the chemical composition of the nanostructures.

## **Cytotoxicity of the prepared Nanosheets**

B16F10 cells (5 × 10³ cells per well) were seeded in 96‐well plates and cultured in DMEM supplemented with 10% FBS and 1% penicillin/streptomycin at 37 °C in a humidified 5% CO₂ atmosphere for 24 h. Subsequently, MnO₂ NS and MnO₂-PEI NS were added at concentrations ranging from 0 to 50 µg/mL, and the cells were incubated at 37 °C for an additional 72 h. Cell viability was evaluated using the CellTiter-Glo® Luminescent Cell Viability Assay (Promega). Briefly, an equal volume of the CellTiter-Glo reagent was added to each well, gently mixed, and incubated at room temperature for 10 min to stabilize the luminescent signal. Luminescence was measured using a Promega’s GloMax Discover System (Promega), and relative cell viability was determined by normalizing the luminescence of treated wells to that of the non-treated control group.

## **Evaluation of ROS generation**

The intracellular reactive oxygen species (ROS) levels were assessed using the DCFDA/H2DCFDA cellular ROS assay kit. B16F10 cells were seeded in 12-well plates and allowed to adhere. After incubation, cells were treated with MnO₂ NS and MnO₂-PEI NS at various concentrations. ROS generation was monitored at multiple time points (0, 2, 4, 6, 24, 48, and 72 h) using the assay kit according to the manufacturer's instructions. Fluorescence intensity was measured to evaluate ROS production over time.

## **Preparation and Characterization of AAV/MnO_2_-PEI NS complex**

To characterize the physicochemical properties of the AAV/MnO₂-PEI NS complex, AAV (1 × 10¹⁰ genome copy) was mixed with MnO₂-PEI NS at various genome copy (gc) to weight ratios. The mixture was incubated at room temperature for 30 min. The size and zeta potential of the complexes were then analyzed using Zetasizer NS90.

## **Gel retardation assay**

To evaluate the encapsulation efficiency of the AAV/MnO₂-PEI NS complex, AAV (1 × 10¹⁰ genome copy) was mixed with MnO₂-PEI NS at various genome copy (gc) to weight ratios. The mixture was incubated at room temperature for 30 min. And the mixtures were treated with virus lysis buffer and incubated at 56 °C for 30 min. Following incubation, each sample was loaded onto a 1% (w/v) agarose gel in 1X TAE buffer containing ethidium bromide and electrophoresed at 100 V for 20 min in the same buffer. Finally, viral DNA bands were visualized using a ChemiDoc gel documentation system.

## **Transduction Efficiency Analysis**

To evaluate the transduction efficiency of the AAV/MnO₂-PEI NS complex, B16F10 cells were seeded in a 24-well plate at a density of 1 × 10⁴ cells per well. GFP-expressing AAV was complexed with MnO₂-PEI NS at various concentrations and incubated at room temperature for 30 minutes. After incubation, AAV alone or various weight ratio of AAV/MnO_2_-PEI NS complexes were treated to the cells at a final concentration of 1 × 10¹⁰ gc per well. GFP expression was assessed 72 h post-transduction, and the percentage of GFP-positive cells was quantified using ImageJ software.

## **Sample Preparation for SAXS measurement**

Solution samples were transferred into a quartz capillary (o.d. 1.5 mm, Hilgenberg, Germany) and flame-sealed using portable torch. The sealed capillary was then measured using custom-built SAXS with the help of Forvis Tech (Santa Barbara, CA). X-ray source had 1.54 Å with an average beam size of 1 mm × 1 mm (V × H). The sample-to-detector distance was 1.55 m, and all samples were measured at room temperature with an exposure time of 3600 s. The scattered photons were recorded using a hybrid pixel detector (Eiger 1M, Dectris, Swiss) and were radially integrated using FIT2D software(http://www.esrf.eu/computing/scientific/FIT2D) from ESRF.

## **SAXS Data Analysis for Diameter Estimation**

Guinier analysis was performed on the SAXS data for the AAV/MnO₂–PEI NS complex at a gc-to-weight ratio of 2.5, yielding a radius of gyration (R_g_) of 13.3 nm. To estimate the overall size of the complex, we employed a spherical shell model, which considers the AAV as a hollow sphere with a shell thickness of approximately 5 nm. The radius of gyration for a spherical shell with an outer radius *R_out_* and inner radius *R_in_* is given by:

$$R_{g}^{2}= \frac{3}{5} \times\frac{R_{out}^{5}- R_{in}^{5}}{R_{out}^{3}- R_{in}^{3}}$$

Since the shell is composed of a lipid bilayer, we can approximate $R_{in}$ as $R_{out}-5 nm$. Solving this equation for *R_out_* using *R_g_* = 13.3 nm, we obtain an outer radius of approximately 15.41 nm, corresponding to a diameter of 30.8 nm. This result supports the nanosheet-wrapped morphology of the AAV/MnO₂–PEI NS complex.

## **Evaluation of cell killing ability**

Cells were seeded into 96-well plates at an appropriate density (5 × 10³ cells/well) and allowed to attach overnight. The next day, cells were divided into four groups: Non-treated control (G1), MnO₂–PEI NS (G2), AAV (G3), and AAV/MnO₂–PEI NS (G4). Treatments were prepared in serum-containing medium and applied to the cells according to each group’s condition. After 72 h of incubation with the respective treatments, cell viability was assessed using the CellTiter-Glo® Luminescent Cell Viability Assay (Promega) according to the manufacturer’s instructions. Luminescence was measured using a microplate reader GloMax® (Promega), and relative cell viability was calculated by normalizing to the non-treated control group (G1). All experiments were performed in triplicate unless otherwise stated.

## **Immunofluorescence**

B16F10 cells were seeded on glass coverslips and treated with four groups (G1–G4). After 72 h, cells were washed with PBS and fixed with 4% paraformaldehyde for 15 min at room temperature. Cells were then permeabilized/blocked with 0.1% Triton X-100 and 5% BSA in PBS for 1 h. Primary staining was performed overnight at 4°C with Anti-Calreticulin (CRT) antibody at a 1:200 dilutions. After washing with PBS, cells were incubated for 1 h at room temperature with an anti-rabbit IgG Alexa Fluor® 488 secondary antibody. Nuclei were counterstained using ProLong™ Gold Antifade Mountant with DNA Stain DAPI. Fluorescent images were acquired at 400× magnification using an Olympus FLUOVIEW FV3000 confocal microscope, and images were processed with the manufacturer’s software. Fluorescence images were quantitatively analyzed by ImageJ software

## **Western blot**

B16F10 cells were seeded in 6-well plates at a density of 2 × 10⁵ cells per well and allowed to stabilize for 24 h. Following stabilization, each well was treated with fresh serum-free medium containing one of the formulations (G1–G4) and incubated for an additional 72 h. Cells were then washed three times with PBS. Proteins were extracted by lysing the cells with RIPA buffer. Protein concentrations were determined using a BCA assay kit with bovine serum albumin as a standard, and 15 µg of protein per sample was subjected to electrophoresis on a 10% polyacrylamide gel. After electrophoretic separation, proteins were transferred to polyvinylidene difluoride (PVDF) membranes. The membranes were incubated with primary antibodies against RIPK3, phospho-RIPK3, MLKL, phosphor-MLKL, or GAPDH, followed by HRP-conjugated anti-rabbit secondary antibody. Protein bands were visualized using an enhanced chemiluminescent reagent.

## **Quantitative RT-PCR**

B16F10 cells were seeded in 6-well plates at a density of 2 × 10⁵ cells per well and allowed to stabilize for 24 h. Following stabilization, each well was treated with fresh serum-free medium containing one of the formulations (G1–G4) and incubated for an additional 72 h. Total RNA was extracted by TRIzol reagent. One microgram of RNA was reverse transcribed into cDNA using a Maxime RT PreMix kit according to the manufacturer’s instructions. To quantify mRNA expression, real-time quantitative PCR analysis was performed using the amfiSure qGreen Q-PCR Master Mix low ROX. Primers used are listed in Table S1. Data were analyzed using the 2^−ΔΔCt^ method and normalized to glyceraldehyde-3-phosphate dehydrogenase (GAPDH) expression.

## **Quantification of intracellular lipid peroxidation**

To assess the induction of lipid oxidation level in cell, B16F10 cells were seeded in 6-well plate at a density of 2 × 10⁵ cells per well and allowed to stabilize for 24 h. Following stabilization, each well was treated with fresh serum-free medium containing one of the formulations (G1–G4) and incubated for an additional 72 h. The cells were homogenized on ice and centrifuged at 12000 rpm for 10 min at 4 °C. The supernatant was used to measure lipid peroxidation using the EZ-lipid peroxidation (TBARS) assay kit according to the manufacturer's instructions and absorbance was recorded at 540 nm.

## **Biodistribution Analysis of AAV/MnO2-PEI NS complex**

The in vivo biodistribution of the AAV/MnO₂-PEI complex was evaluated using a B16F10 tumor model. This model was established by injecting 5 × 10⁵ B16F10 cancer cells into the right flank of C57BL/6 mice. When the tumor volume reached approximately 200 mm³, mice bearing tumors received an intravenous injection of one of the three formulations: (1) Cy5 only, (2) MnO₂-PEI-Cy5, and (3) AAV/MnO₂-PEI-Cy5. MnO₂-PEI NS was conjugated with Cy5 via a reaction with Cy5 NHS prior to injection. In vivo fluorescence imaging was performed using an IVIS system at 0.5, 1, 3, and 24 h post-injection to monitor Cy5 intensity. At 24 h, the mice were euthanized, and major organs were harvested for ex vivo imaging. The average fluorescence intensity of each organ was quantified to assess the biodistribution of the formulations.

## **Safety evaluation**

To evaluate the in vivo toxicity of each formulation, mice (n = 3 per group) were intravenously injected with 1 × 10¹¹ gc of AAV, MnO₂-PEI NS, or AAV/MnO₂-PEI NS once every two days for a total of three injections, with PBS serving as the control. Two days after the final injection, the mice were euthanized, and major organs (liver, lung, heart, kidney, and spleen) were harvested for H&E staining. Additionally, serum levels of aspartate aminotransferase (AST), alanine transaminase (ALT), blood urea nitrogen (BUN), and creatinine (CREA) were measured.

## **Antitumor effect**

B16F10 cells (5 × 10⁵) suspended in 50 µL of HBSS were subcutaneously injected into the right flank of C57BL/6 mice to establish a subcutaneous tumor model. Tumor volume was calculated using the formula: Volume = 0.523 × Length × (Width)². Once the tumors reached approximately 70 mm³, mice were intravenously administered one of the following formulations: non-treated (G1), MnO₂-PEI NS (G2), AAV (G3), or AAV/MnO₂-PEI NS (G4). Treatments were performed every two days for a total of three injections.

## **Histological and immunohistochemical analyses**

At the end of the tumor inhibition study, tumors were excised from sacrificed mice and fixed in a 4% formaldehyde solution, followed by paraffin embedding. The embedded tumors were sectioned into 10 µm-thick slices and stained with hematoxylin and eosin to evaluate the therapeutic effect by light microscopy at 200× magnification. Additionally, apoptotic cells in the collected tumors were analyzed using a terminal deoxynucleotidyl transferase dUTP nick-end labeling (TUNEL) assay kit according to the manufacturer's protocol. Tumor sections were also stained with anti-Ki67 or anti-RIPK3 antibodies to assess cancer cell proliferation and RIPK3 expression, respectively. The slides were imaged using confocal laser scanning microscopy at 400x magnification. TUNEL positive cells in tissue and the expression levels of Ki-67 and RIPK3 were quantitatively assessed using the ImageJ software. Data are expressed as percentage of green fluorescent positive area of three different images.

## **FACS**

B16F10 tumor-bearing C57BL/6 mice were treated with four groups (G1–G4) every two days for a total of three injections. Three days after the final injection, tumors were excised, transferred to a dish, and cut into small pieces (<1 mm³). The tissue fragments were suspended in 1 mL of digestion solution (0.3 mg/mL type IV collagenase and 0.15 mg/mL deoxyribonuclease I in DMEM containing 10% FBS) and incubated at 37 °C for 1 h with continuous agitation. Cells were then collected by centrifugation at 400 g for 5 min and filtered through a 100 μm cell strainer. Finally, cells were stained with an Fc receptor blocker (CD16/32) and fluorescence-labeled antibodies, and the following cell populations were analyzed by flow cytometer: M1 macrophages (CD45⁺ CD11b^+^ F4/80⁺ CD80⁺), M2 macrophages (CD45⁺ CD11b^+^ F4/80⁺ CD206⁺), Mature dendritic cells (CD45⁺ CD11c⁺ CD80⁺ CD86⁺), and CD8⁺ T cells (CD45⁺ CD3⁺ CD8⁺).

## **ELISA**

Intratumoral levels of IFN-γ and CXCL-10 were quantified using specific ELISA kits. Briefly, tumor tissues from each treatment group were collected and homogenized. The homogenates were then centrifuged, and the cytokine levels in the supernatants were measured using a mouse IFN-γ ELISA assay kit and a mouse CXCL-10 ELSIA assay kit.

## **Establishment of re-challenge tumor model and efficacy test**

B16F10 cells (5 × 10⁵ cells per mouse) were suspended in HBSS buffer and subcutaneously injected into C57BL/6 mice. The mice were then randomly assigned to different treatment groups. Following the final injection after 2 days, the primary tumors were surgically resected, and B16F10 cells were re-injected into the opposite flank of the mice to establish a re-challenge model. Tumor size in the re-challenge model was measured using the following formula: Volume = 0.523 × Length × (Width)². After 21 days of re-challenge tumor inoculation, tumor tissues and spleens were harvested, digested, and dissociated into single-cell suspensions. The cells were then stained with conjugated antibodies, including anti-CD3-BV510, anti-CD8a-BV711, anti-CD62L-PE/Cyanine7, anti-CD44L-PE/Cyanine5. The stained cells were analyzed by flow cytometry.

## **Statistical analysis**

All data were analyzed statistically using GraphPad Prism 8 software. Results are expressed as the mean ± standard deviation (SD). The statistical significance of the data was assessed using one-way analysis of variance (ANOVA). The p-values of * p < 0.05, ** p < 0.01, and *** p < 0.001 were considered statistically significant.

**Table S1.** Primer sequences

| Gene | Primer sequence |
| --- | --- |
| GPX4 | F : 5’- GAT GGA GCC CAT TCC TGA ACC-3’  R : 5’- CCC TGT ACT TAT CCA GGC AGA-3’ |
| FPN | F : 5’ – TGG GTG GAT AAG AAT GCC -3’  R : 5’ – ATG ATC CCG CAG AGA ATG-3’ |
| GAPDH | F : 5′-AGA ACA TCA TCC CTG CAT CC-3′  R : 5′-ACA CAT TGG GGG TAG GAA CA-3′ |
| Survivin | F : 5′-TGG CAG CTG TAC CTC AAG AA-3′  R : 5′-AGC TGC TCA ATT GAC TGA CG-3′ |

**Reference**

[1]Z. Liu, K. Xu, H. Sun, S. Yin, One-Step Synthesis of Single-Layer MnO2 Nanosheets with Multi-Role Sodium Dodecyl Sulfate for High-Performance Pseudocapacitors, Small, 2015, 11 (18), 2182.

**
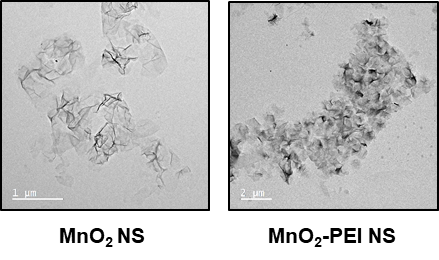
**

**Figure S1.** TEM image of MnO_2_ NS and MnO_2_-PEI NS. Scale bar represents 1 μm and 2 μm respectively.


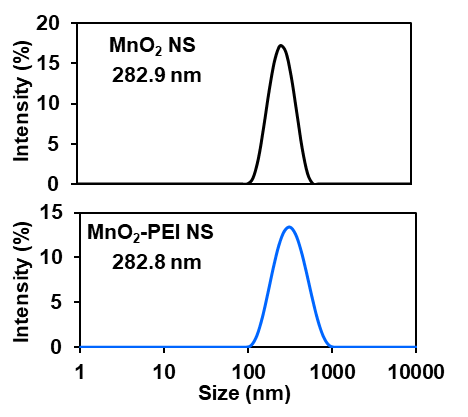


**Figure S2.** Hydrodynamic size distribution of MnO_2_ NS and MnO_2_-PEI NS.

**
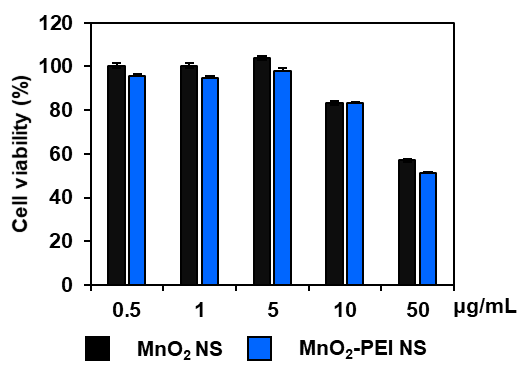
**

**Figure S3.** Cell cytotoxicity of MnO_2_ NS and MnO_2_-PEI NS (n = 3). Data are presented as mean ± SD.


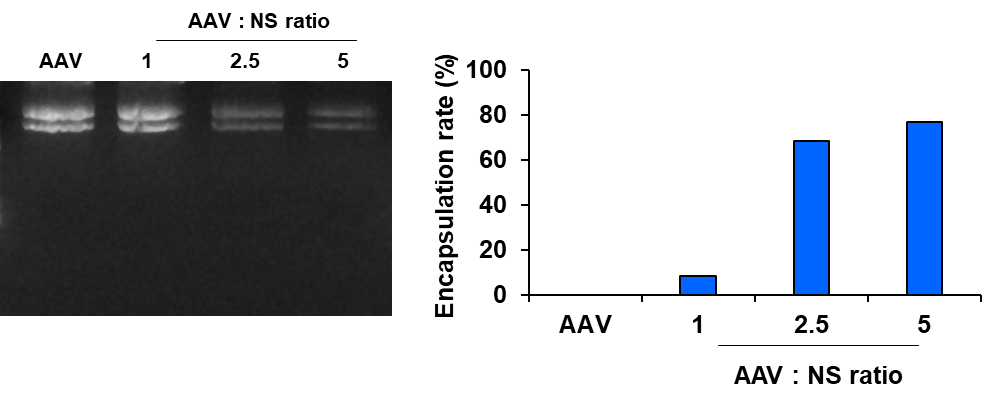


**Figure S4.** Gel retardation assay and encapsulation rate of AAV/MnO_2_-PEI

**Figure S5.** SAXS analysis of AAV.


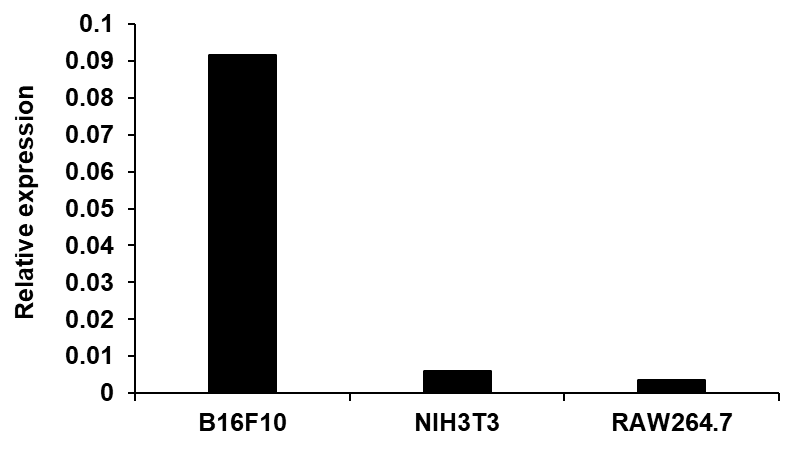


**Figure S6.** Relative survivin expression level in various cell lines (n = 3). Data are presented as mean ± SD.

**
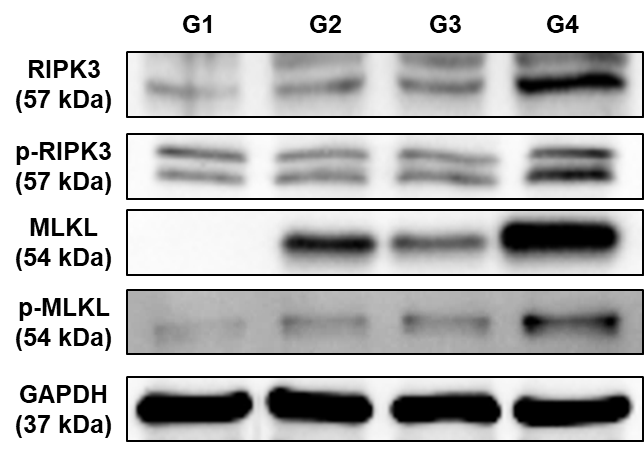
**

**Figure S7.** Representative immunoblot image of indicated proteins in B16F10 cells after treated with different formulations.


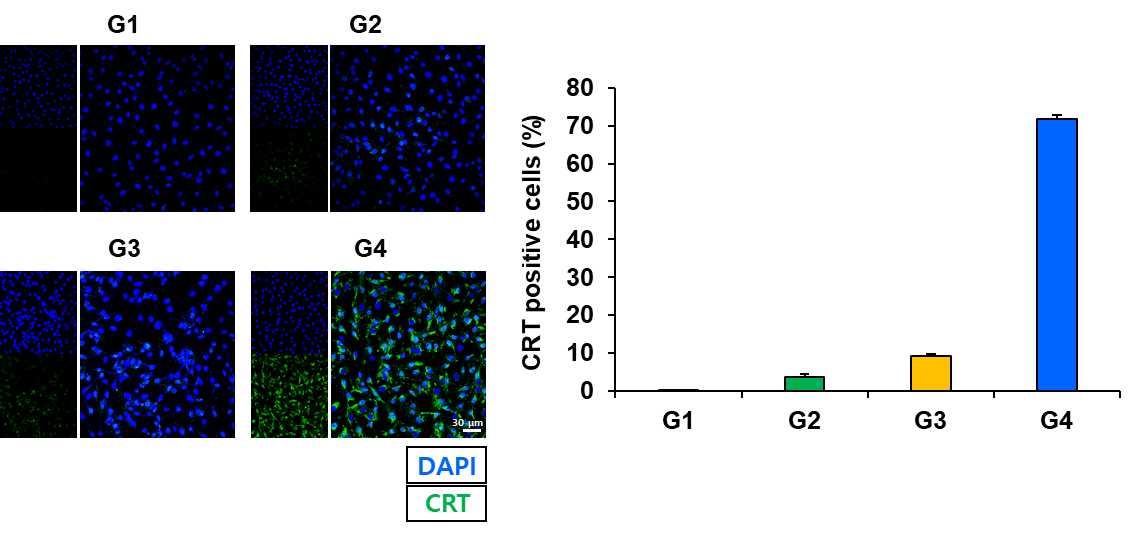


**Figure S8.** Immunofluorescence image of CRT in B16F10 cells after incubation with different formulations for 72 h. CRT positive cells were quantified by Image J software (n = 3). Data are presented as mean ± SD. Scale bar is 30 μm.


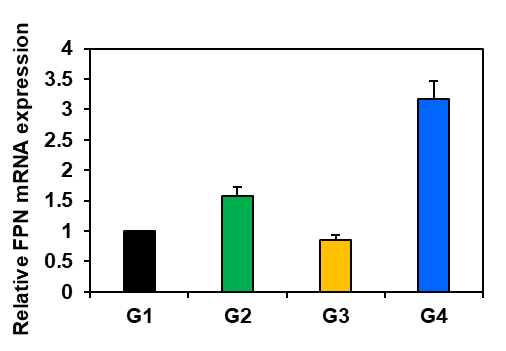


**Figure S9.** Relative ferroportin (FPN) mRNA expression level (n = 3). Data are presented as mean ± SD.

**
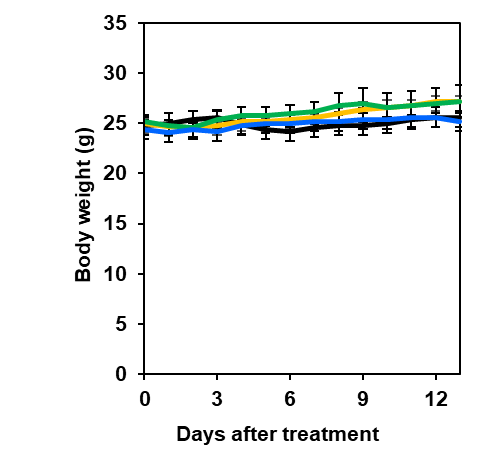
**

**Figure S10.** Body weights of tumor-bearing mice during treatment (n = 5). Data are presented as mean ± SD.

**
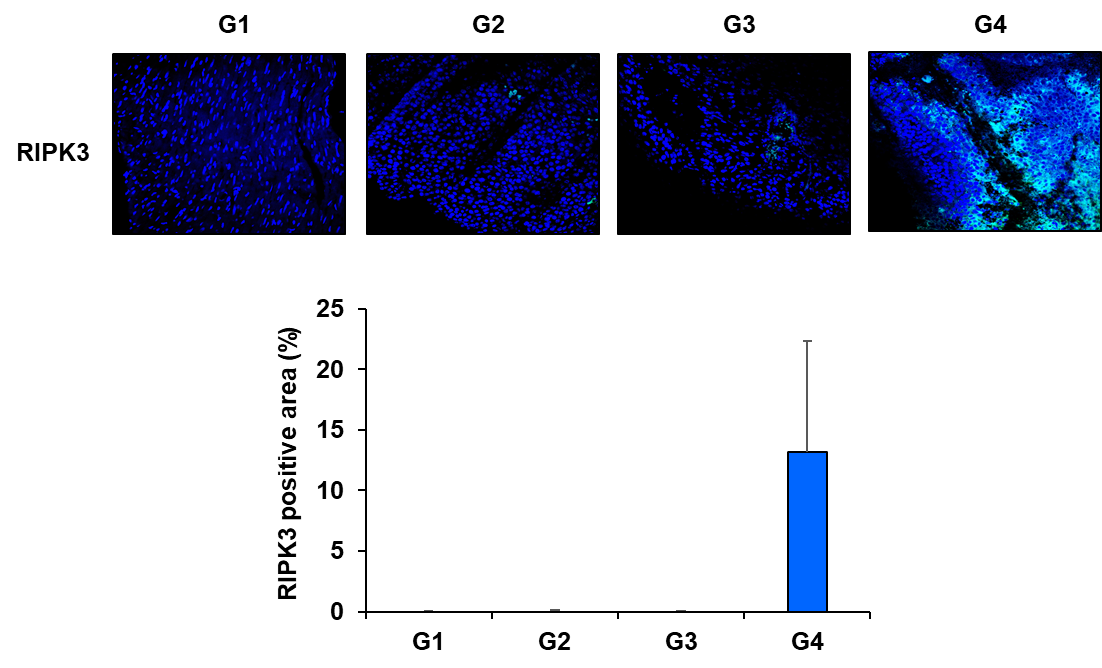
**

**Figure S11.**  RIPK3 antigen staining of tumor tissues harvested from corresponding 3 days after the administration of the last treatment. RIPK3 positive cells were quantified by Image J software (n = 3). Data are presented as mean ± SD. Scale bar is 100 μm.


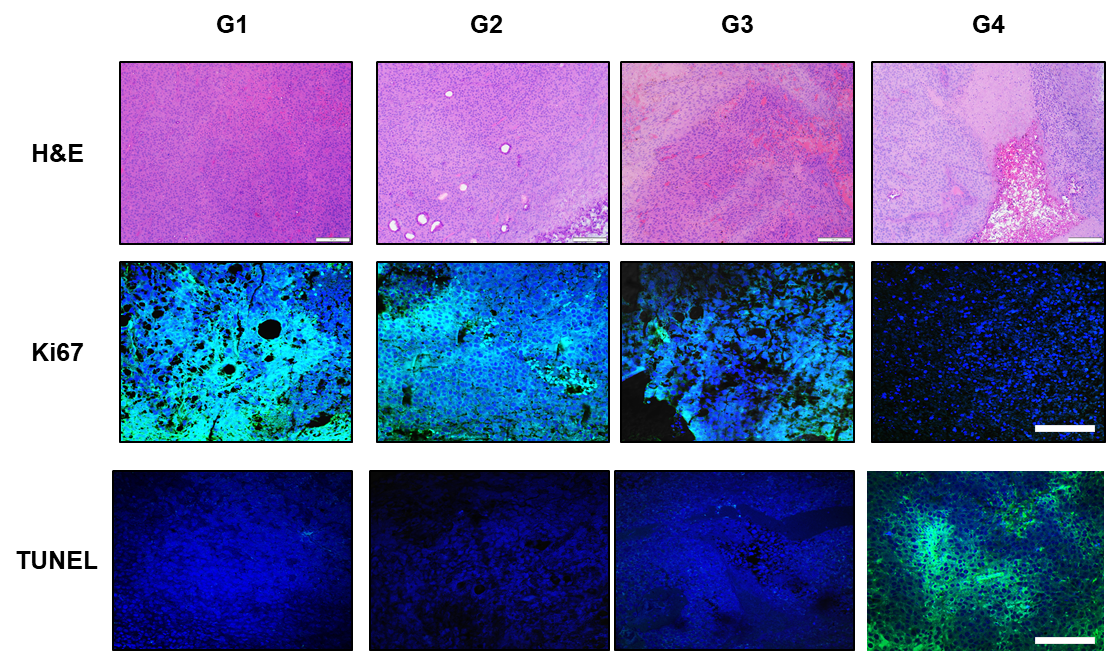


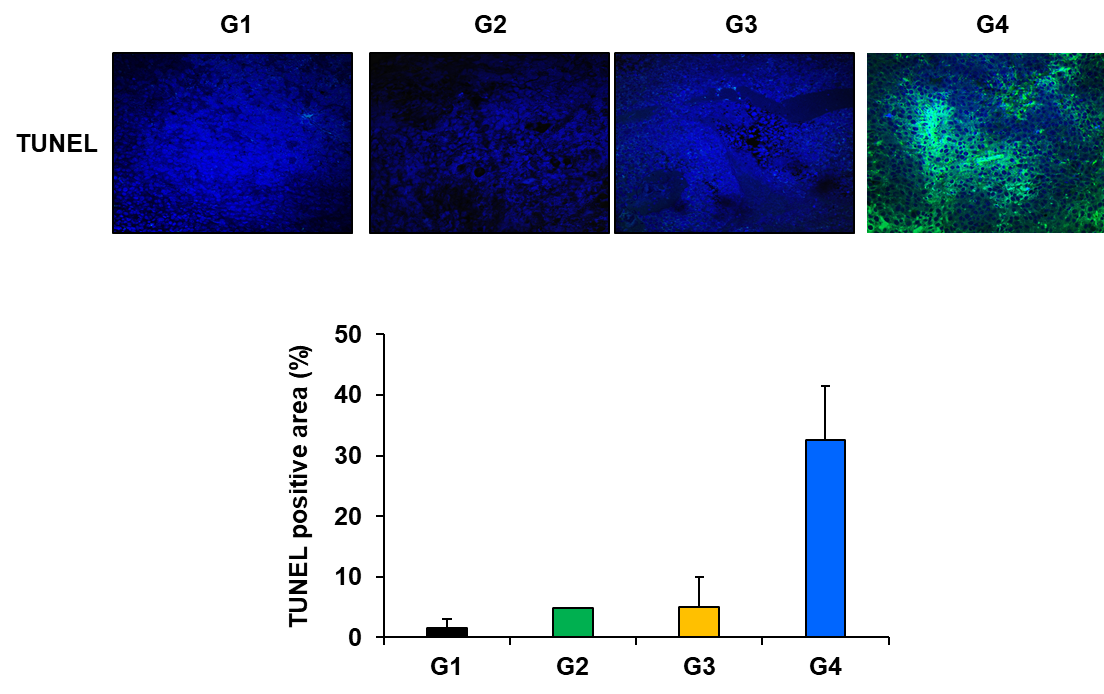


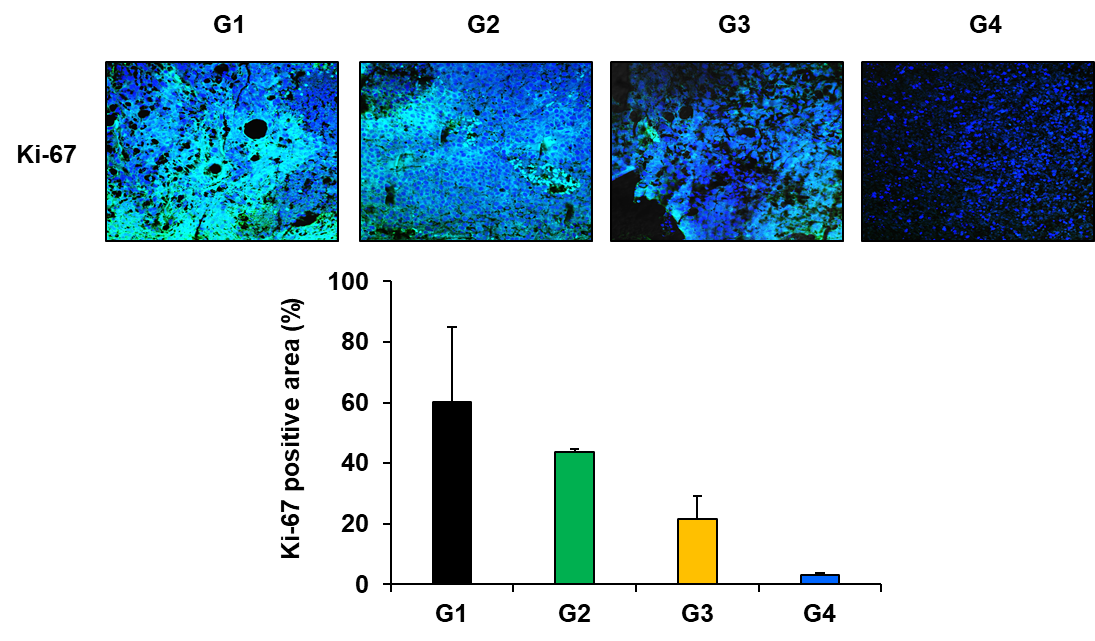


**Figure S12.** H&E, TUNEL and Ki-67 antigen staining of tumor tissues harvested from corresponding mice after 13 days of treatments. TUNEL positive and Ki-67 positive cells were quantified by Image J software (n = 3). Data are presented as mean ± SD. Scale bar is 100 μm.


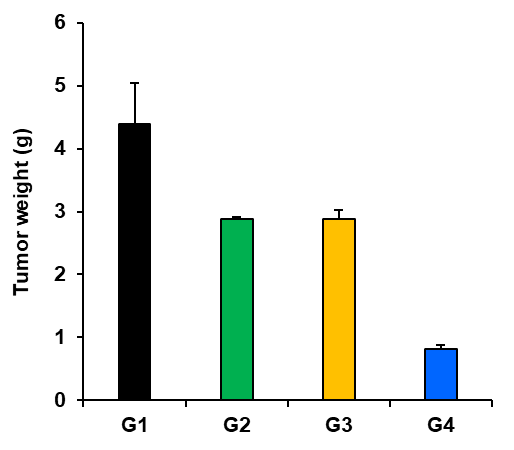


**Figure S13.** The tumor weights 21 days after re-challenge tumor inoculation (n = 3). Data are presented as mean ± SD.
